# Clarithromycin and dexamethasone show similar anti-inflammatory effects on distinct phenotypic chronic rhinosinusitis: an explant model study

**DOI:** 10.1186/s12865-015-0096-x

**Published:** 2015-06-06

**Authors:** Ming Zeng, Zhi-Yong Li, Jin Ma, Ping-Ping Cao, Heng Wang, Yong-Hua Cui, Zheng Liu

**Affiliations:** Department of Otolaryngology-Head and Neck Surgery, Tongji Hospital, Tongji Medical College; Huazhong University of Science and Technology, No. 1095 Jiefang Avenue, Wuhan, 430030 People’s Republic of China

**Keywords:** Chronic rhinosinusitis, Nasal polyps, Clarithromycin, Dexamethasone, Eosinophil, Inflammation, Tissue remodeling, Innate immunity

## Abstract

**Background:**

Phenotype of chronic rhinosinusitis (CRS) may be an important determining factor of the efficacy of anti-inflammatory treatments. Although both glucocorticoids and macrolide antibiotics have been recommended for the treatment of CRS, whether they have different anti-inflammatory functions for distinct phenotypic CRS has not been completely understood. The aim of this study is to compare the anti-inflammatory effects of clarithromycin and dexamethasone on sinonasal mucosal explants from different phenotypic CRS ex vivo.

**Methods:**

Ethmoid mucosal tissues from CRSsNP patients (n = 15), and polyp tissues from eosinophilic (n = 13) and non-eosinophilic (n = 12) CRSwNP patients were cultured in an ex vivo explant model with or without dexamethasone or clarithromycin treatment for 24 h. After culture, the production and/or expression of anti-inflammatory molecules, epithelial-derived cytokines, pro-inflammatory cytokines, T helper (Th)1, Th2 and Th17 cytokines, chemokines, dendritic cell relevant markers, pattern recognition receptors (PRRs), and tissue remodeling factors were detected in tissue explants or culture supernatants by RT-PCR or ELISA, respectively.

**Results:**

We found that both clarithromycin and dexamethasone up-regulated the production of anti-inflammatory mediators (Clara cell 10-kDa protein and interleukin (IL)-10), whereas down-regulated the production of Th2 response and eosinophilia promoting molecules (thymic stromal lymphopoietin, IL-25, IL-33, CD80, CD86, OX40 ligand, programmed cell death ligand 1, CCL17, CCL22, CCL11, CCL5, IL-5, IL-13, and eosinophilic cationic protein) and Th1 response and neutrophilia promoting molecules (CXCL8, CXCL5, CXCL10, CXCL9, interferon-γ, and IL-12), from sinonasal mucosa from distinct phenotypic CRS. In contrast, they had no effect on IL-17A production. The expression of PRRs (Toll-like receptors and melanoma differentiation-associated gene 5) was induced, and the production of tissue remodeling factors (transforming growth factor-β1, epidermal growth factor, basic fibroblast growth factor, platelet derived growth factor, vascular endothelial growth factor, and matrix metalloproteinase 9) was suppressed, in different phenotypic CRS by dexamethasone and clarithromycin in comparable extent.

**Conclusions:**

Out of our expectation, our explant model study discovered herein that glucocorticoids and macrolides likely exerted similar regulatory actions on CRS and most of their effects did not vary by the phenotypes of CRS.

## Background

Chronic rhinosinusitis (CRS) is a group of heterogeneous inflammatory disorders of nose and the paranasal sinuses. Based on the presence or absence of nasal polyps, CRS is classified into CRS with nasal polyps (CRSwNP) and CRS without nasal polyps (CRSsNP) [1]. Eosinophilic inflammation has been considered to be a cardinal feature of CRSwNP in whites for a long time. However, in Asians, only half of CRSwNP present eosinophilic inflammation, indicating a more heterogeneous feature of CRSwNP in Asians [2, 3]. Although the etiology of CRS remains enigmatic, all phenotypic CRS are characterized by prolonged and persistent inflammation in the lesional sinonasal mucosa [1, 2]. Therefore, the anti-inflammatory treatment is currently considered as a primary treatment for CRS [1, 4].

Glucocorticoids have been widely used to control CRS given their powerful and broad anti-inflammatory effects [4, 5]. Glucocorticoids can suppress the chemotaxis and activation of various immune cells including eosinophils, T cells, and mast cells, etc. [5]. They can induce apoptosis of eosinophils and suppress the release of an array of inflammatory cytokines, chemokines, and mediators from resident and inflammatory cells in tissues [5]. Beyond the well-known antimicrobial effect, increasing evidences have emerged to show that macrolides have intrinsic anti-inflammation and immunomodulation properties [6, 7]. Macrolides can block the activation of transcription factor nuclear factor κB and inhibit the production of various inflammatory cytokines, including interleukin (IL)-8 and tumor necrosis factor-α (TNF-α) [6, 7]. They can also suppress the secretion of airway mucus, induce the apoptosis of neutrophils, and even diminish the formation of bacterial biofilms [6, 7]. Some studies have demonstrated the in vivo effect of long-time, low-dose macrolide treatment on controlling CRS [1, 8–10].

Although glucocorticoids and macrolides have been recommended for the treatment of CRS by European Position Paper on Rhinosinusitis and Nasal Polyps [1], there are a number of CRS patients that do not response well to glucocorticoid treatment and conflicting results exist regarding the efficacy of macrolide treatment in CRS [1, 9, 10]. The reasons for the variations of efficacy of glucocorticoids and macrolides are unclear, but part of the problem is heterogeneity of CRS, with several different pathways contributing to disease in different patients. In whites, CRSsNP presents a predominant T helper (Th) 1 milieu, whereas CRSwNP is characterized by a Th2-skewed eosinophilic inflammation [3]. Nevertheless, in Chinese, only eosinophilic, but not non-eosinophilic, CRSwNP demonstrates a Th2-dominated inflammation [2, 11]. In addition, Th17 responses that are almost absent in white patients with CRS have been found up-regulated in Chinese [2, 3, 11–13]. Our recent study has shown that although oral prednisone is able to suppress the Th2-dominated eosinophilic inflammation, it cannot inhibit the Th17 responses and associated neutrophilic inflammation in Chinese patients with CRSwNP [14]. Wallwork et al. have found that macrolides may only be efficient for CRSsNP patients without elevated serum IgE levels [9]. We have found that long-term clarithromycin treatment could inhibit IL-8 and myeloperoxidase production in Chinese patients with CRSsNP and clarithromycin was more effective for CRSsNP patients with high levels of IL-8 [15]. These studies suggest that the phenotype of CRS might be a potential determining factor of the efficacy of anti-inflammation agents, and glucocorticoids and macrolides might prefer to control eosinophilic and neutrophilic inflammation, respectively. However, the effects of glucocorticoids and macrolides on the inflammatory responses in distinct phenotypic CRS have not been carefully and comprehensively compared. Hence, in this study we compared the effects of dexamethasone and clarithromycin on different inflammatory pathways in sinonasal mucosa from Chinese patients with CRSsNP, and eosinophilic and non-eosinophilic CRSwNP by using an ex vivo tissue explant culture model.

## Results

### The pilot study of dose response effect of clarithromycin and dexamethasone

**Fig. 1:**
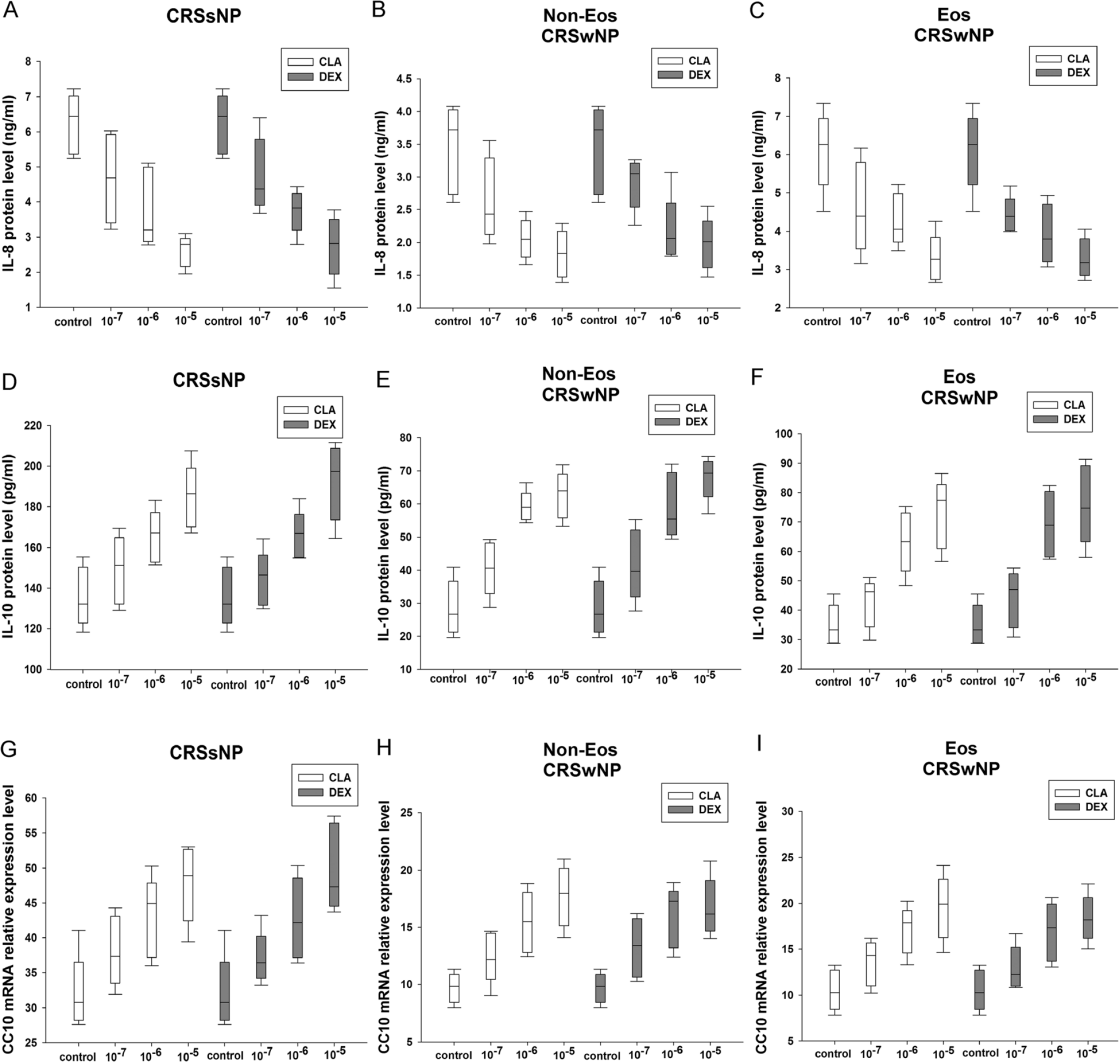
The concentration-dependent effect of dexamethasone and clarithromycin on the production of interleukin (IL)-8, IL-10 and Clara cell 10-kD protein (CC10). Both of dexamethasone and clarithromycin demonstrated a significant concentration-dependent effect on the protein production of (**a**-**c**) IL-8 and (**d**-**f**) IL-10 and (**g**-**i**) mRNA production of CC10 from sinonasal mucosa from different phenotypic chronic rhinosinusitis after 24-h culture (*P* < 0.05). Maximal effect was seen at a concentration of 10^−5^ mol/L. An equivalent volume of methanol solution was used as control for dexamethasone and clarithromycin. CRSsNP, chronic rhinosinusitis without nasal polyps; Non-Eos CRSwNP, non-eosinophilic chronic rhinosinusitis with nasal polyps; Eos CRSwNP, eosinophilic chronic rhinosinusitis with nasal polyps; CLA, clarithromycin; DEX, dexamethasone. n = 5 for CRSsNP, and Eos and Non-Eos CRSwNP group

**Fig. 2:**
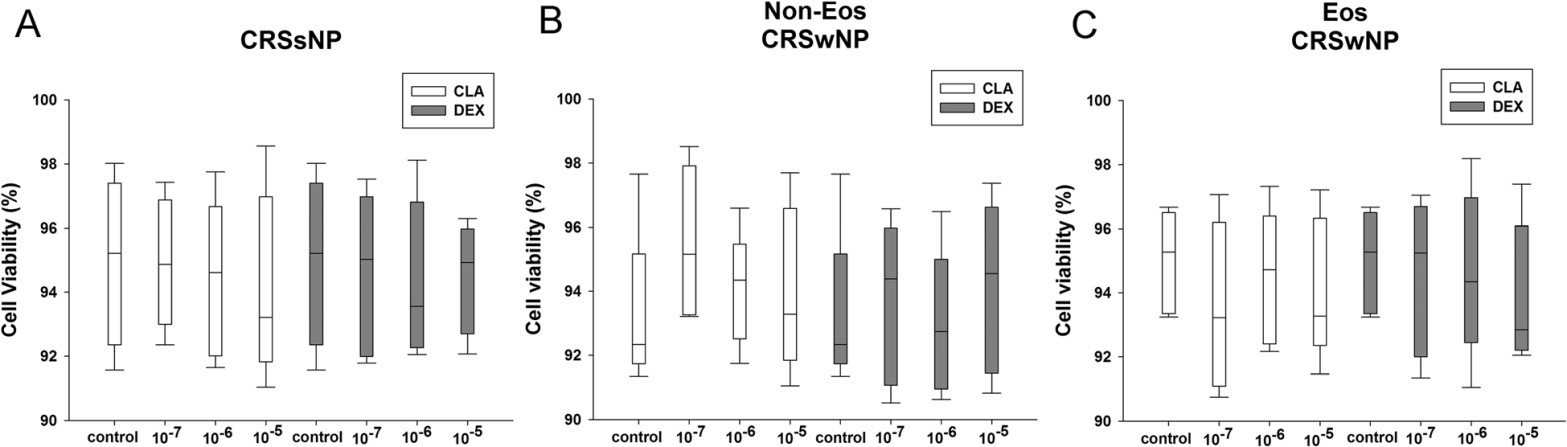
The tissue cell viability determined by trypan blue dye exclusion test. Dexamethasone and clarithromycin demonstrated no significant influence on the viability of tissue cells from different phenotypic chronic rhinosinusitis at serial concentrations of 10^−7^ mol/L, 10^−6^ mol/L, and 10^−5^ mol/L, as compared to controls after 24-h tissue explant culture. An equivalent volume of methanol solution was used as control for dexamethasone and clarithromycin. CRSsNP, chronic rhinosinusitis without nasal polyps; Non-Eos CRSwNP, non-eosinophilic chronic rhinosinusitis with nasal polyps; Eos CRSwNP, eosinophilic chronic rhinosinusitis with nasal polyps; CLA, clarithromycin; DEX, dexamethasone. n = 5 for CRSsNP, and Eos and Non-Eos CRSwNP group

**Fig. 3:**
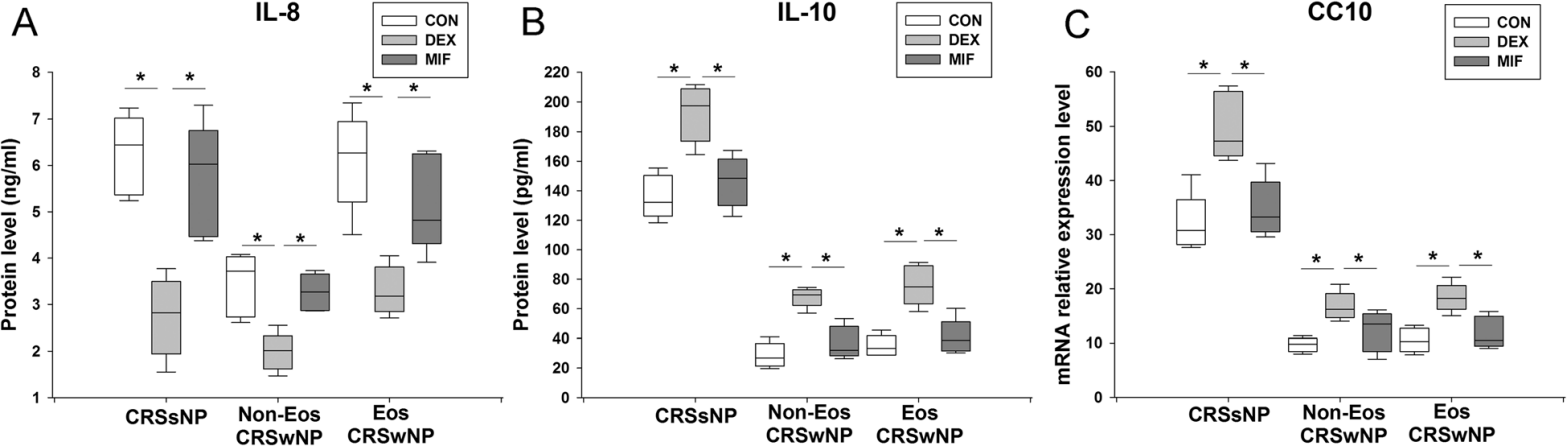
Glucocorticoid receptor antagonist, mifepristone, diminishes the effect of dexamethasone. Mifepristone (10^−5^ mol/L) antagonized the effect of dexamethasone (10^−5^ mol/L) on the reduction of (**a**) IL-8 protein, and the promotion of (**b**) IL-10 protein and (**c**) Clara cell 10-kD protein (CC10) mRNA production in the sinonasal mucosa from different phenotypic chronic rhinosinusitis after 24-h culture. An equivalent volume of methanol solution was used as control for dexamethasone and clarithromycin. CRSsNP, chronic rhinosinusitis without nasal polyps; Non-Eos CRSwNP, non-eosinophilic chronic rhinosinusitis with nasal polyps; Eos CRSwNP, eosinophilic chronic rhinosinusitis with nasal polyps; CLA, clarithromycin; DEX, dexamethasone; MIF, mifepristone. n = 5 for CRSsNP, and Eos and Non-Eos CRSwNP group. *P < 0.05

In the pilot experiments, we found a concentration-dependent effect of clarithromycin and dexamethasone on the reduction of IL-8, and induction of IL-10 and Clara cell 10-kD protein (CC10) production in sinonasal mucosa from CRSsNP, and eosinophilic and non-eosinophilic CRSwNP with a maximal response at a concentration of 10^−5^ mol/L (*P* < 0.05) (Fig. 1). In contrast, we found that compared with controls, dexamethasone and clarithromycin at serial concentrations of 10^−7^ mol/L, 10^−6^ mol/L, and 10^−5^ mol/L did not have significant influence on tissue cell viability after 24-h culture, and viability of tissue cells from all CRS groups was >91 % in all experiment conditions (Fig. 2). In order to confirm the specific effect of dexamethasone at a relatively high concentration of 10^−5^ mol/L, a glucocorticoid receptor antagonist, mifepristone, was added to the culture. We discovered that mifepristone could diminish the effect of 10^−5^ mol/L of dexamethasone on IL-8, IL-10, and CC10 production in sinonasal mucosa from all three CRS groups, confirming the specific effect of dexamethasone (Fig. 3).

### The effects of dexamethasone and clarithromycin on epithelial-derived mediators

**Fig. 4:**
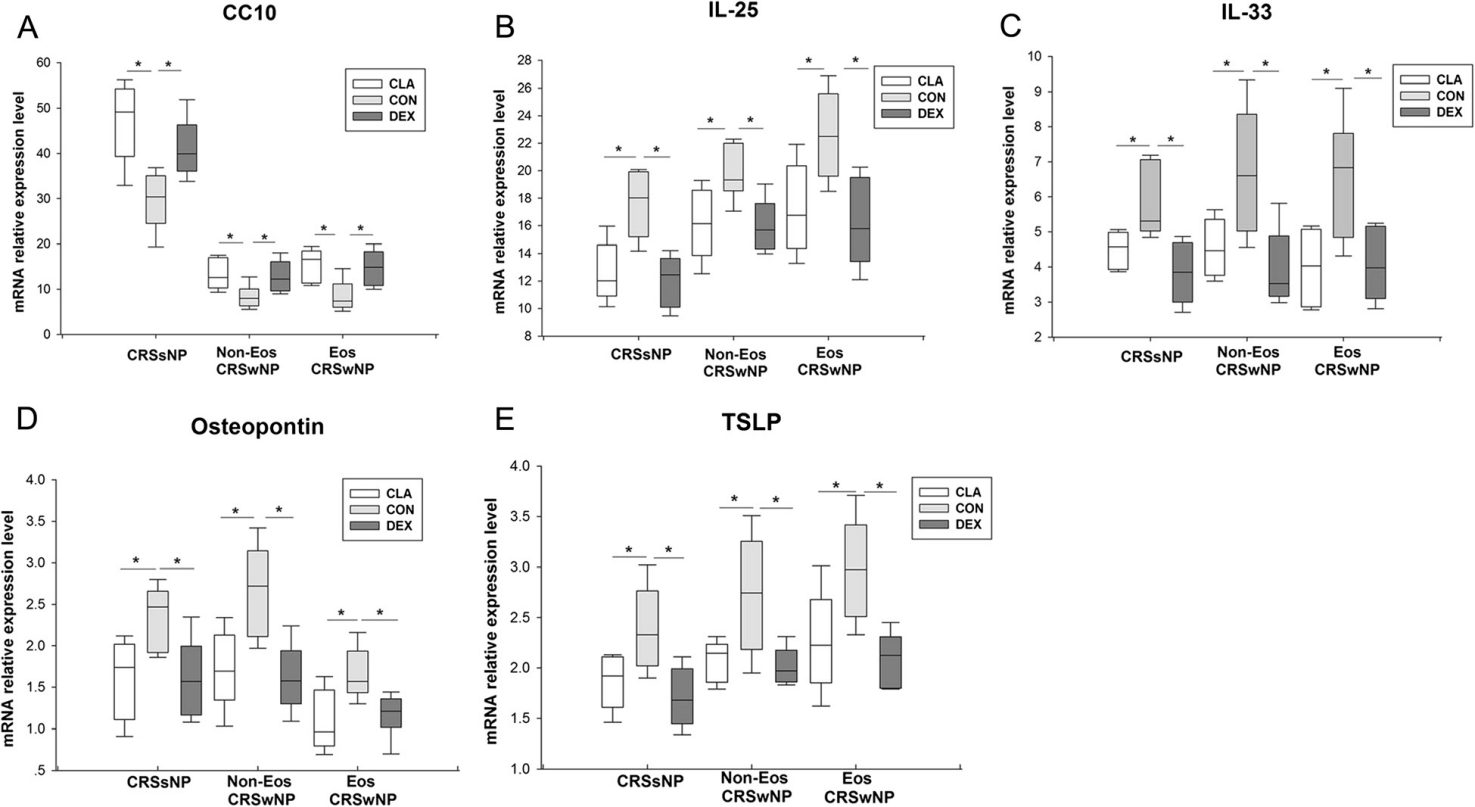
The effect of dexamethasone and clarithromycin on the expression of epithelial-derived mediators. The mRNA relative expression of (**a**) Clara cell 10-kD protein (CC10), (**b**) interleukin (IL)-25, (**c**) IL-33, (**d**) osteopontin, and (**e**) thymic stromal lymphopoietin (TSLP) in the sinonasal mucosa from different phenotypic chronic rhinosinusitis after 24-h culture with 10^−5^ mol/L of dexamethasone or clarithromycin. An equivalent volume of methanol solution was used as control for dexamethasone and clarithromycin. CRSsNP, chronic rhinosinusitis without nasal polyps; Non-Eos CRSwNP, non-eosinophilic chronic rhinosinusitis with nasal polyps; Eos CRSwNP, eosinophilic chronic rhinosinusitis with nasal polyps; CLA, clarithromycin; CON, control; DEX, dexamethasone. n = 5 for CRSsNP group; n = 6 for Eos and Non-Eos CRSwNP group. *P < 0.05

Our pilot experiments showed that 10^−5^ mol/L of dexamethasone and clarithromycin had maximal pharmaceutical effect without affecting the tissue cell viability. In addition, this concentration was reported comparable to that seen in serum during oral administration of clarithromycin, and was close to local concentration when glucocorticoids are delivered intranasally, respectively [16, 17]. Thus, we have chosen 10^−5^ mol/L as the concentration for the following comparison experiment. We found that dexamethasone and clarithromycin could similarly up-regulate the mRNA expression of CC10 (Fig. 4a), whereas down-regulate the mRNA expression of IL-25 (Fig. 4b), IL-33 (Fig. 4c), osteopontin (Fig. 4d) and thymic stromal lymphopoietin (TSLP) (Fig. 4e), in sinonasal mucosa from CRSsNP, and eosinophilic and non-eosinophilic CRSwNP patients.

### The effects of dexamethasone and clarithromycin on pro-inflammatory cytokines

**Fig. 5:**
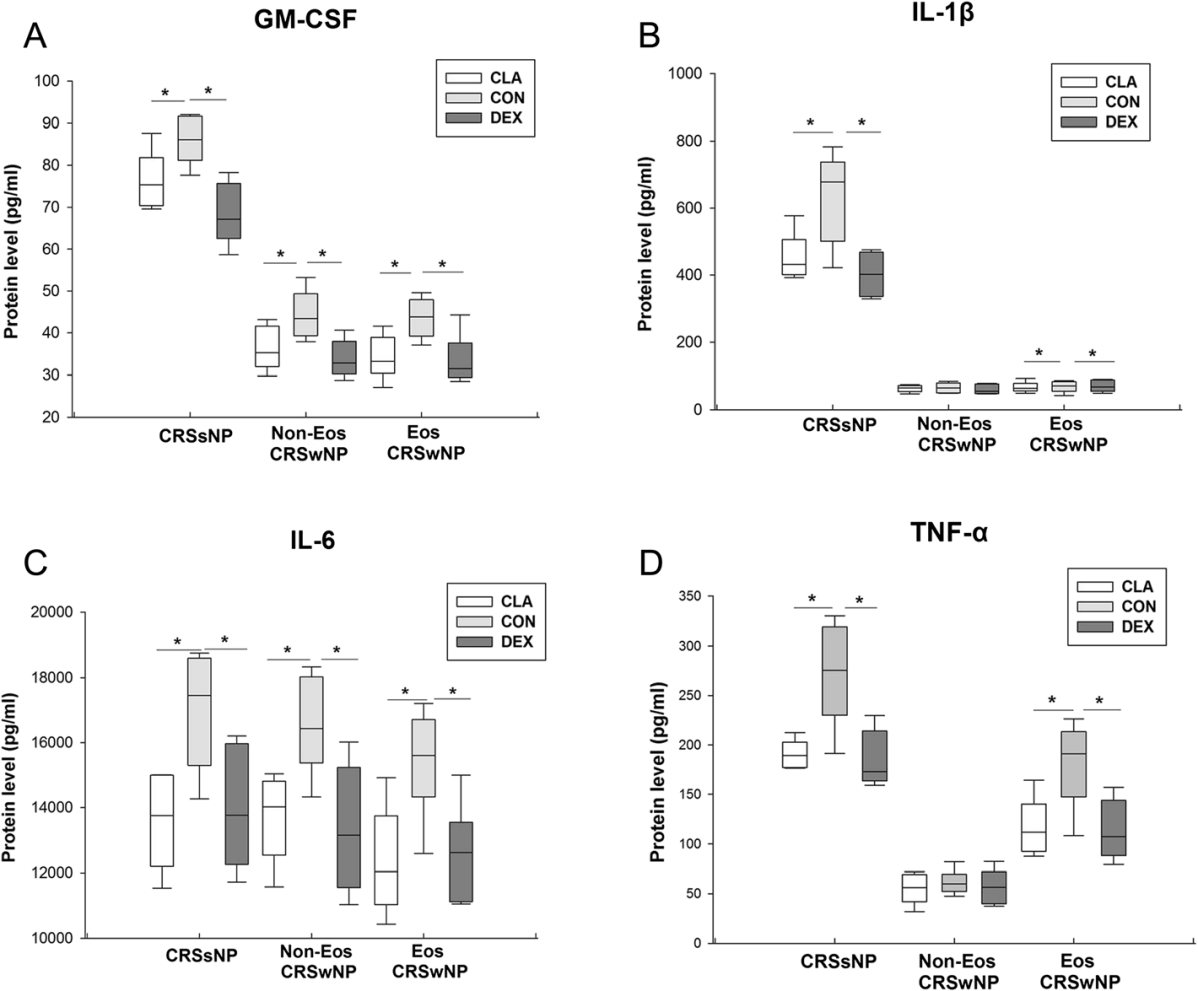
The effect of dexamethasone and clarithromycin on the production of pro-inflammatory cytokines. The protein levels of (**a**) granulocyte-macrophage colony stimulating factor (GM-CSF), (**b**) interleukin (IL)-1β, (**c**) IL-6, and (**d**) tumor necrosis factor-α (TNF-α) in culture supernatants from different phenotypic chronic rhinosinusitis after 24-h culture with 10^−5^ mol/L of dexamethasone or clarithromycin. An equivalent volume of methanol solution was used as control for dexamethasone and clarithromycin. CRSsNP, chronic rhinosinusitis without nasal polyps; Non-Eos CRSwNP, non-eosinophilic chronic rhinosinusitis with nasal polyps; Eos CRSwNP, eosinophilic chronic rhinosinusitis with nasal polyps; CLA, clarithromycin; CON, control; DEX, dexamethasone. n = 5 for CRSsNP group; n = 6 for Eos and Non-Eos CRSwNP group. *P < 0.05

Both dexamethasone and clarithromycin inhibited the protein production of granulocyte-macrophage colony stimulating factor (GM-CSF) (Fig. 5a) and IL-6 (Fig. 5c) from sinonasal mucosa from all three CRS groups in similar extent. They could also suppress the protein production of IL-1β (Fig. 5b) and tumor necrosis factor (TNF)-α (Fig. 5d) from sinonasal mucosa from CRSsNP and eosinophilic CRSwNP patients, but not from non-eosinophilic CRSwNP patients.

### The effect of dexamethasone and clarithromycin on chemokines

**Fig. 6:**
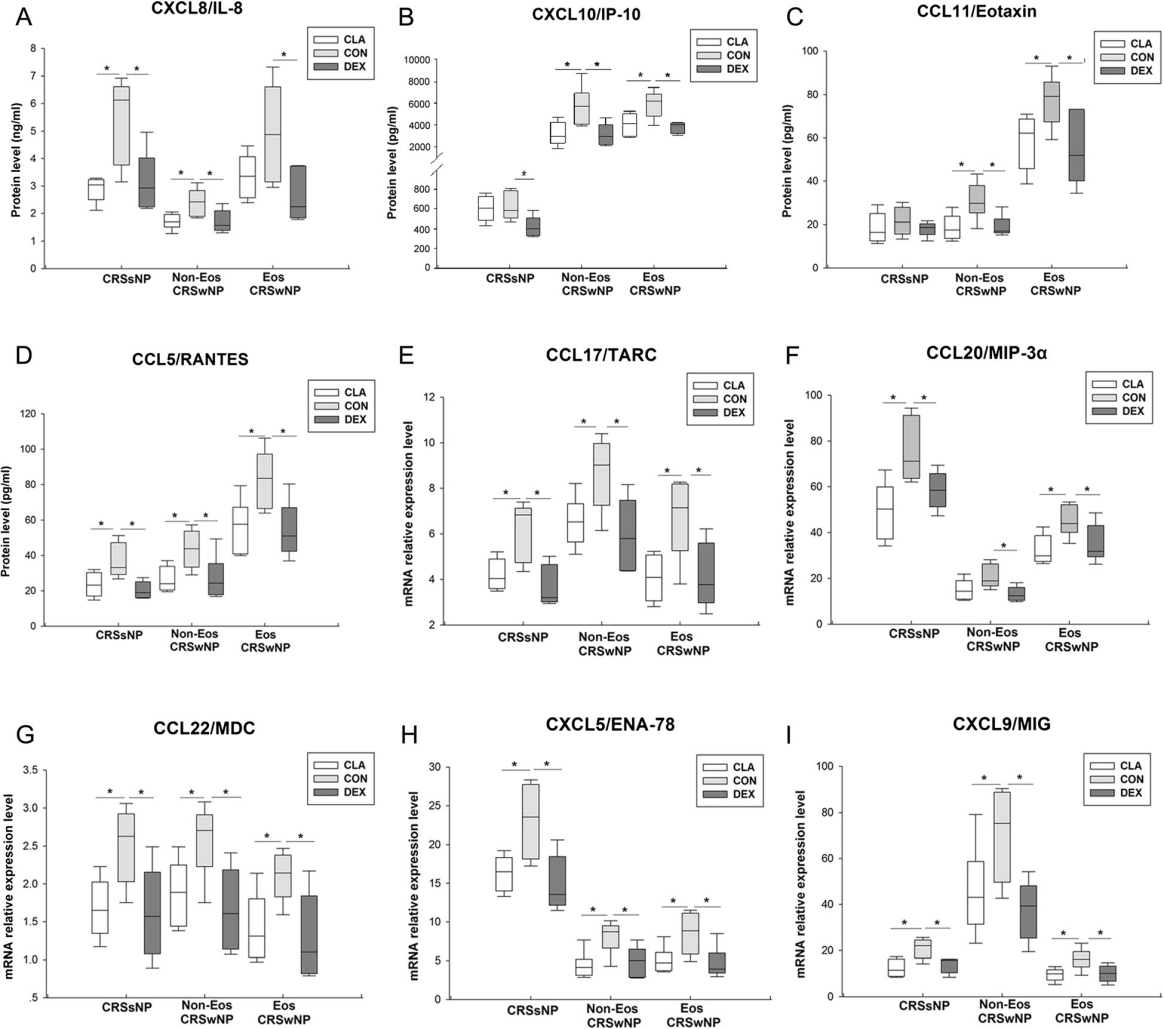
The effect of dexamethasone and clarithromycin on the production or expression of chemokines. The protein levels of (**a**) CXC chemokine ligand (CXCL) 8/interleukin (IL)-8, (**b**) CXCL10/interferon-γ-induced protein 10 (IP-10), (**c**) CC chemokine ligand (CCL)11/eotaxin and (**d**) CCL5/regulated upon activation normal T cell expressed and secreted (RANTES) in culture supernatants, and mRNA expression levels of (**e**) CCL17/thymus and activation-regulated chemokine (TARC), (**f**) CCL20/macrophage inflammatory protein-3α (MIP-3α), (**g**) CCL22/macrophage-derived chemokine (MDC), (**h**) CXCL5/epithelial neutrophil-activating peptide-78 (ENA-78) and (**i**) CXCL9/monokine induced by IFN-γ (MIG) in sinonasal mucosa, from different phenotypic chronic rhinosinusitis after 24-h culture with 10^−5^ mol/L of dexamethasone or clarithromycin. An equivalent volume of methanol solution was used as control for dexamethasone and clarithromycin. CRSsNP, chronic rhinosinusitis without nasal polyps; Non-Eos CRSwNP, non-eosinophilic chronic rhinosinusitis with nasal polyps; Eos CRSwNP, eosinophilic chronic rhinosinusitis with nasal polyps; CLA, clarithromycin; CON, control; DEX, dexamethasone. n = 5 for CRSsNP group; n = 6 for Eos and Non-Eos CRSwNP group. *P < 0.05

Dexamethasone and clarithromycin suppressed the protein levels of CC chemokine ligand (CCL) 5/regulated upon activation normal T cell expressed and secreted (RANTES) (Fig. 6d) in culture supernatants, and mRNA expression of CCL17/thymus and activation-regulated chemokine (TARC) (Fig. 6e), CCL22/macrophage-derived chemokine (MDC) (Fig. 6g), CXC chemokine ligand (CXCL) 5/epithelial neutrophil-activating peptide-78 (ENA-78) (Fig. 6h) and CXCL9/monokine induced by interferon-γ (MIG) (Fig. 6i) in sinonasal mucosa, from CRSsNP and eosinophilic and non-eosinophilic CRSwNP patients in similar extent. Clarithromycin and dexamethasone could also similarly decrease the protein production of CXCL8/IL-8 (Fig. 6a) from CRSsNP and non-eosinophilic CRSwNP and CXCL10/interferon-γ-induced protein 10 (IP-10) (Fig. 6b) and CCL11/eotaxin (Fig. 6c) from eosinophilic and non-eosinophilic CRSwNP, and the mRNA expression of CCL20/macrophage inflammatory protein-3α (MIP-3α) (Fig. 6f) in CRSsNP and eosinophilic CRSwNP. In addition, dexamethasone, but not clarithromycin, statistically significantly down-regulated CXCL8/IL-8 protein levels (Fig. 6a) in culture supernatants from eosinophilic CRSwNP, CXCL10/IP-10 protein levels (Fig. 6b) in culture supernatants from CRSsNP, and mRNA expression levels of CCL20/MIP-3α (Fig. 6f) in sinonasal mucosa from non-eosinophilic CRSwNP.

### The effect of dexamethasone and clarithromycin on DC relevant markers

**Fig. 7:**
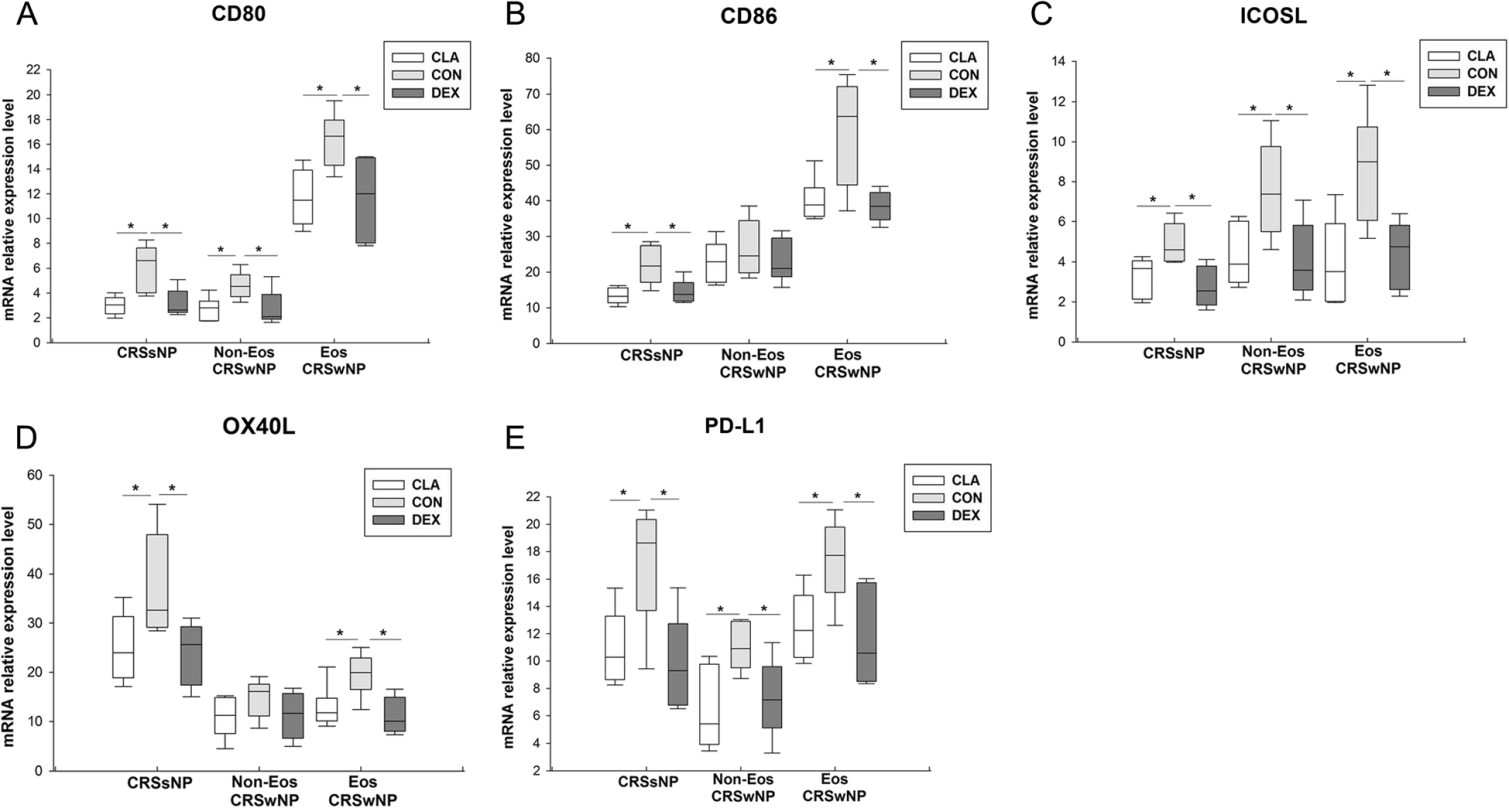
The effect of dexamethasone and clarithromycin on the expression of dendritic cell relevant markers. The mRNA expression of (**a**) CD80, (**b**) CD86, (**c**) inducible costimulator ligand (ICOSL), (**d**) OX40 ligand (OX40L), and (**e**) programmed cell death ligand 1 (PD-L1) in sinonasal mucosa from different phenotypic chronic rhinosinusitis after 24-h culture with 10^−5^ mol/L of dexamethasone or clarithromycin. An equivalent volume of methanol solution was used as control for dexamethasone and clarithromycin. CRSsNP, chronic rhinosinusitis without nasal polyps; Non-Eos CRSwNP, non-eosinophilic chronic rhinosinusitis with nasal polyps; Eos CRSwNP, eosinophilic chronic rhinosinusitis with nasal polyps; CLA, clarithromycin; CON, control; DEX, dexamethasone. n = 5 for CRSsNP group; n = 6 for Eos and Non-Eos CRSwNP group. *P < 0.05

Dexamethasone and clarithromycin could comparably down-regulate the mRNA expression of CD86 (Fig. 7b) and OX40 ligand (OX40L) (Fig. 7d) in sinonasal mucosa from CRSsNP and eosinophilic CRSwNP patients, whereas no statistically significant effect was observed for sinonasal mucosa from non-eosinophilic CRSwNP patients. Both dexamethasone and clarithromycin could down-regulate the mRNA expression of CD80 (Fig. 7a), inducible costimulator ligand (ICOSL) (Fig. 7c) and programmed cell death ligand 1 (PD-L1) (Fig. 7e) in sinonasal mucosa from all three CRS groups in similar extent.

### The effect of dexamethasone and clarithromycin on Th cytokines

**Fig. 8:**
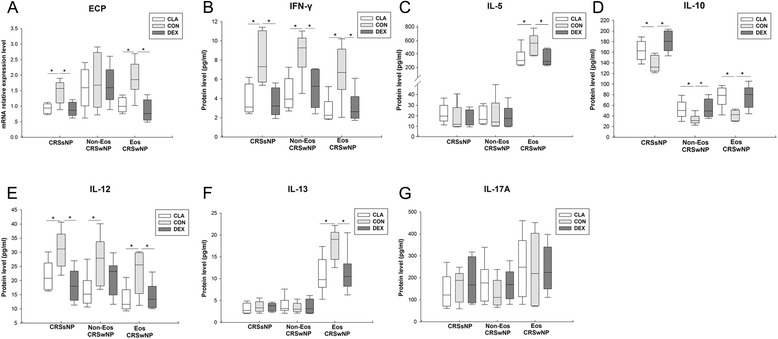
The effect of dexamethasone and clarithromycin on production or expression of Th cytokines. The mRNA relative expression levels of (**a**) eosinophilic cationic protein (ECP) in sinonasal mucosa, and the protein levels of (**b**) interferon (IFN)-γ, (**c**) interleukin (IL)-5, (**d**) IL-10, (**e**) IL-12, (**f**) IL-13, and (**g**) IL-17A in culture supernatants, from different phenotypic chronic rhinosinusitis after 24-h culture with 10^−5^ mol/L of dexamethasone or clarithromycin. An equivalent volume of methanol solution was used as control for dexamethasone and clarithromycin. CRSsNP, chronic rhinosinusitis without nasal polyps; Non-Eos CRSwNP, non-eosinophilic chronic rhinosinusitis with nasal polyps; Eos CRSwNP, eosinophilic chronic rhinosinusitis with nasal polyps; CLA, clarithromycin; CON, control; DEX, dexamethasone. n = 5 for CRSsNP group; n = 6 for Eos and Non-Eos CRSwNP group. *P < 0.05

After treatment with dexamethasone or clarithromycin, we detected a remarkable and similar decrease of interferon (IFN)-γ protein production (Fig. 8b) from sinonasal mucosa from all CRS groups and IL-12 protein production (Fig. 8e) from sinonasal mucosa from CRSsNP and eosinophilic CRSwNP. Clarithromycin also suppressed IL-12 production (Fig. 8e) from sinonasal mucosa from non-eosinophilic CRSwNP. Dexamethasone and clarithromycin could significantly inhibit the protein production of IL-5 (Fig. 8c) and IL-13 (Fig. 8f) in only eosinophilic polyp tissues, possibly due to the very low expression levels of IL-5 and IL-13 in CRSsNP and non-eosinophilic CRSwNP. In contrast, neither dexamethasone nor clarithromycin had significant effect on IL-17A (Fig. 8g) protein production in CRS. In addition, both dexamethasone and clarithromycin inhibited the mRNA expression of eosinophilic cationic protein (ECP) (Fig. 8a) in sinonasal mucosa from CRSsNP and eosinophilic CRSwNP, but not in those from non-eosinophilic CRSwNP. On the contrary, we observed a significantly and comparably up-regulated production of IL-10 (Fig. 8d) protein from sinonasal mucosa from all CRS groups after dexamethasone or clarithromycin treatment.

### The effect of dexamethasone and clarithromycin on the PRRs

**Fig. 9:**
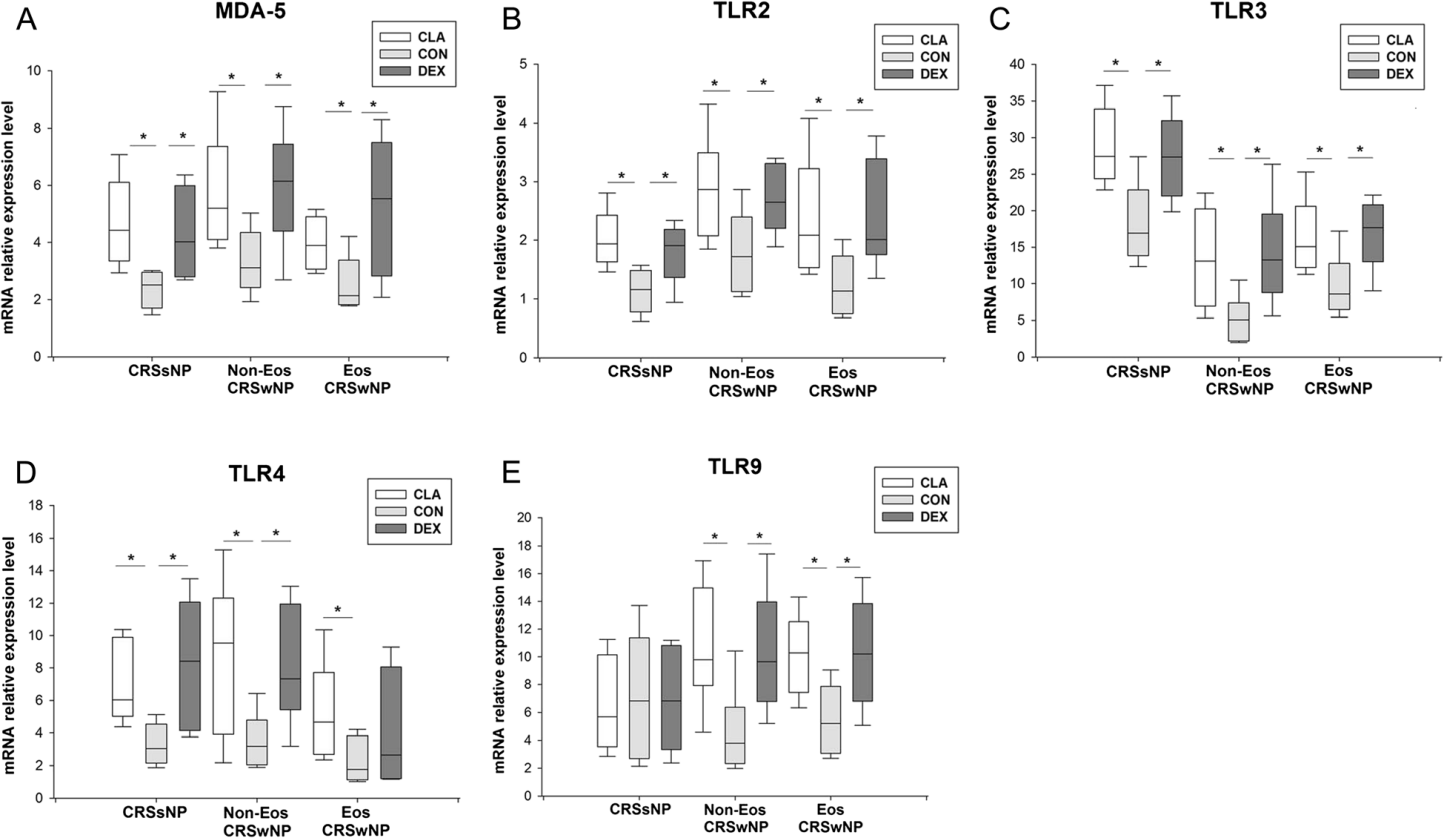
The effect of dexamethasone and clarithromycin on the expression of pattern recognition receptors. The mRNA expression levels of (**a**) melanoma differentiation-associated gene-5 (MDA-5), (**b**) toll like receptor (TLR)2, (**c**) TLR3, (**d**) TLR4, and (**e**) TLR9 in the sinonasal mucosa from different phenotypic chronic rhinosinusitis after 24-h culture with 10^−5^ mol/L of dexamethasone or clarithromycin. An equivalent volume of methanol solution was used as control for dexamethasone and clarithromycin. CRSsNP, chronic rhinosinusitis without nasal polyps; Non-Eos CRSwNP, non-eosinophilic chronic rhinosinusitis with nasal polyps; Eos CRSwNP, eosinophilic chronic rhinosinusitis with nasal polyps; CLA, clarithromycin; CON, control; DEX, dexamethasone. n = 5 for CRSsNP group; n = 6 for Eos and Non-Eos CRSwNP group. *P < 0.05

Interestingly, we found that both dexamethasone and clarithromycin were able to increase the mRNA expression of melanoma differentiation-associated gene 5 (MDA-5) (Fig. 9a), toll like receptor (TLR) 2 (Fig. 9b) and TLR3 (Fig. 9c) in sinonasal mucosa from all CRS groups, and mRNA expression of TLR9 (Fig. 9e) in polyp tissues from eosinophilic and non-eosinophilic CRSwNP patients in similar extent. Clarithromycin could up-regulate the mRNA expression of TLR4 in sinonasal mucosa from all CRS groups; while dexamethasone might enhance the TLR4 expression in sinonasal mucosa from CRSsNP and non-eosinophilic CRSwNP (Fig. 9d).

### The effect of dexamethasone and clarithromycin on tissue remodeling factors

**Fig. 10:**
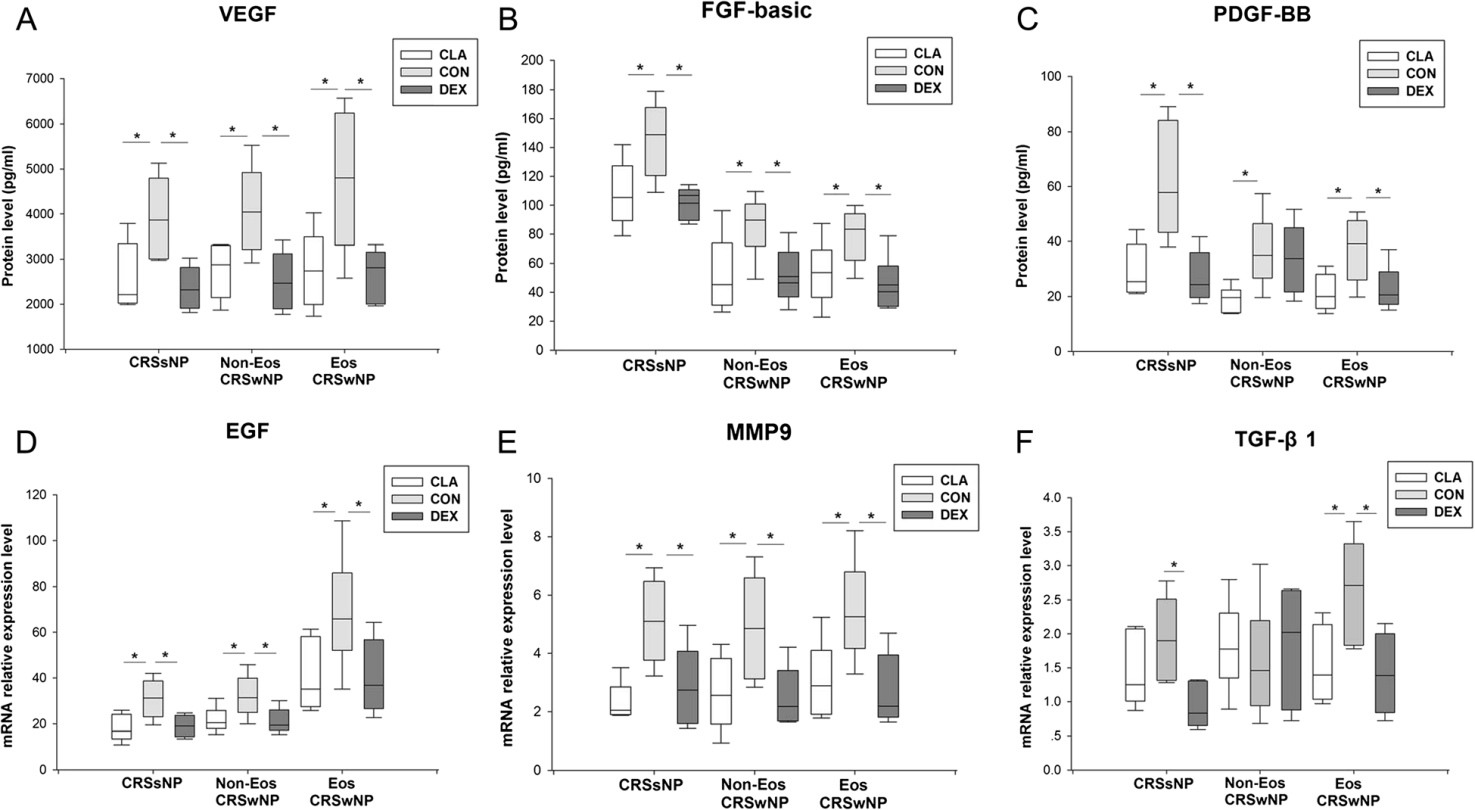
The effect of dexamethasone and clarithromycin on the expression or production of tissue remodeling factors. The protein levels of (**a**) vascular endothelial growth factor (VEGF), (**b**) basic fibroblast growth factor (FGF-basic) and (**c**) platelet derived growth factor-BB (PDGF-BB) in culture supernatants, and the mRNA relative expression levels of (**d**) epidermal growth factor (EGF), (**e**) matrix metalloproteinases 9 (MMP9) and (**f**) transforming growth factor-β1 (TGF-β1) in sinonasal mucosa, from different phenotypic chronic rhinosinusitis after 24-h culture with 10^−5^ mol/L of dexamethasone or clarithromycin. An equivalent volume of methanol solution was used as control for dexamethasone and clarithromycin. CRSsNP, chronic rhinosinusitis without nasal polyps; Non-Eos CRSwNP, non-eosinophilic chronic rhinosinusitis with nasal polyps; Eos CRSwNP, eosinophilic chronic rhinosinusitis with nasal polyps; CLA, clarithromycin; CON, control; DEX, dexamethasone. n = 5 for CRSsNP group; n = 6 for Eos and Non-Eos CRSwNP group. *P < 0.05

The protein production of vascular endothelial growth factor (VEGF) (Fig. 10a) and basic fibroblast growth factor (FGF-basic) (Fig. 10b), and the mRNA production of epidermal growth factor (EGF) (Fig. 10d) and matrix metalloproteinase 9 (MMP9) (Fig. 10e) from sinonasal mucosa from all CRS groups could be down-regulated by dexamethasone and clarithromycin in similar extent. In addition, both dexamethasone and clarithromycin was able to similarly suppress the protein production of platelet derived growth factor-BB (PDGF-BB) (Fig. 10c) from sinonasal mucosa from CRSsNP and eosinophilic CRSwNP patients, and mRNA production of transforming growth factor-β1 (TGF-β1) (Fig. 10f) from sinonasal mucosa from eosinophilic CRSwNP. Clarithromycin could also down-regulate PDGF-BB (Fig. 10c) production in non-eosinophilic CRSwNP, and dexamethasone was capable to decrease TGF-β1 (Fig. 10f) production in CRSsNP.

## Discussion

Development of phenotype-orientated therapeutic strategies is critical for the improvement of CRS treatment. Whether glucocorticoids and macrolides are more effective for specific phenotypic CRS has not been completely understood. In this study, we have comprehensively compared the efficacy of dexamethasone and clarithromycin on the expression and/or production of epithelial-derived mediators, anti- and pro-inflammatory cytokines, chemokines, DC relevant markers, Th1/Th2/Th17 cytokines, PRRs, and tissue remodeling factors in Chinese CRSsNP, and eosinophilic and non-eosinophilic CRSwNP by using a tissue explant culture model. To the best of our knowledge, this is the first study to carefully compare the anti-inflammatory actions of glucocorticoids and macrolides in the different phenotypic CRS. Overall, we are surprised to find that dexamethasone and clarithromycin exerted similar anti-inflammation effects on different inflammatory pathways in CRS and most of their effects did not vary by the phenotypes of CRS in this tissue explant study.

CC10 and IL-10 are two important anti-inflammatory molecules in airways. Our previous studies have shown that CC10 and IL-10 production in sinonasal mucosa can be induced by glucocorticoids [18, 19]. In this study, we not only confirmed effect of dexamethasone which validated our present experimental system, but also demonstrated a similar effect of clarithromycin on CC10 and IL-10 induction in all phenotypic CRS, suggesting both agents may exert their anti-inflammatory function through promoting the production of anti-inflammatory mediators.

“Epithelium-DC-Th cell” cross-talk and chemokines play a key role in the formation of polarized Th response and biased granulocyte activation in sinonasal mucosa. Epithelial-derived TSLP, IL-25 and IL-33 are critical in licensing innate and adaptive immunity and promote Th2 responses [20]. Our recent study has shown that CD86^+^ activated myeloid and plasmacytoid DCs were increased in both eosinophilic and non-eosinophilic CRSwNP [11]. The OX40L/PD-L1^+^ lesional DCs likely under the influence of TSLP in eosinophilic CRSwNP can prime Th2 cells, whereas the low OX40L/PD-L1-expressing lesional DCs with a possible influence by osteopontin in non-eosinophilic CRSwNP primarily induce Th1/Th17 cells [11]. In this study, surprisingly, we found that not only dexamethasone but also clarithromycin was able to suppress the expression of TSLP, IL-25, IL-33, CD80, CD86, OX40L, and PD-L1. Moreover, the expression of Th2 cell and eosinophil chemokines, including CCL17/TARC, CCL22/MDC, CCL11/eotaxin and CCL5/RANTES, was also inhibited by both dexamethasone and clarithromycin. These changes were coinciding with the down-regulation of Th2 cytokines (IL-5 and IL-13) and ECP by both dexamethasone and clarithromycin in eosinophilic polyp, indicating that beyond its well-known suppression on neutrophilic inflammation [15, 21], macrolides may also possess an inhibitory effect on the Th2-dominated eosinophilic inflammation as glucocorticoids. In fact, there are few previous reports showing that macrolides treatment can reduce the ECP levels in nasal secretion from CRS patients [21].

On the other hand, clarithromycin and dexamethasone also demonstrated comparable action on ameliorating neutrophilic inflammation through diminishing the expression of neutrophil chemokines (e.g., CXCL8/IL-8 and CXCL5/ENA-78) in CRS. Moreover, both clarithromycin and dexamethasone suppressed Th1 responses by decreasing the expression of Th1 cell chemokines (CXCL10/IP-10 and CXCL9/MIG) and Th1 cytokines (IFN-γ and IL-12), but had no effect on IL-17A production, in all phenotypic CRS. Consistently, our previous in vivo study has showed that oral prednisone treatment could not suppress Th17 response in Chinese CRSwNP [14]. Thus, these studies arouse the need to seeking novel therapies targeting Th17 responses in CRS.

Pro-inflammatory cytokines act as intercellular signals to regulate the functions of DCs and other immune cells. In this study, we found that GM-CSF, IL-6, TNF-α and IL-1β production could be diminished by dexamethasone and clarithromycin, which is consistent with previous reports and in line with their inhibitory effect on NF-κB signaling pathway [5, 17, 22, 23].

Previously, Zhang et al. have elegantly demonstrated that although glucocorticoids could inhibit acute phase response in airway epithelial cells, it may spare or enhance the expression of local innate host defense molecules, such as complements, collectins, and other antimicrobial proteins [24]. In addition, Homma et al. have found that dexamethasone synergistically increased TLR2 expression in respiratory epithelial cells in combination with TNF-α and IFN-γ [25]. PRRs are crucial in recognizing a wide range of microbial pathogens and triggering signaling cascades that activate effective immune responses. In line with Zhang’s and Homma’s reports [24, 25], interestingly, we found herein that although glucocorticoids and macrolides were able to diminish the expression of an array of inflammatory molecules, they did increase the expression of TLRs and MDA-5 in sinonasal mucosa from CRS. These data suggest that glucocorticoids and macrolides may reinforce the local innate host defense against infectious organisms in airways and therefore reduce the exacerbation of CRS.

Besides the persistent inflammation, the sinonasal mucosa of CRS patients is also characterized by marked tissue remodeling [26, 27]. Our previous study demonstrated distinct remodeling features of different phenotypic CRS in Chinese [26]. Eosinophilic CRSwNP shows marked edema; on the contrary, CRSsNP presents significant fibrosis [26, 27]. This remodeling process may be controlled by growth factor-induced extracellular matrix deposition and proteases-dominated degradation [26–29]. Extending previous findings on dexamethasone [30–32], we found herein that both dexamethasone and clarithromycin might suppress the remodeling process in CRS through suppressing the expression of remodeling relevant mediators.

We have to acknowledge several limitations of our current study that ask us to explain our results with caution. The most important one is that although we used tissue explant culture to simulate in vivo condition as closely as possible, whether these two agents can exert same efficacy in vivo needs further study. Secondly, we treated tissue samples for 24-h that is significant different from the time period of treatment we used in clinic. Thirdly, we did not do the dose–response experiments for all studied parameters given the limited amount of tissue samples. Moreover, it should be noted that our current study is a small sample size study and further confirmation with a larger population is needed. Fourthly, although the concentration of 10^−5^ mol/L we used in tissue explant culture is close to the concentration achievable in patients and both drugs at this concentration did not reduce the tissue cell viability, we still need to explain our data with caution since this concentration, especially for dexamethasone, is much higher than that commonly used in cell culture study. Fifthly, for some molecules, we only measured the mRNA expression levels because of the limited amount of tissue samples, therefore the changes of these molecules at protein level wait to be confirmed in future. Sixthly, although we found the changes of the expression and/or production of these molecules in CRS, the intracellular and intercellular mechanisms underlying the actions of dexamethasone and clarithromycin remain to be defined in future.

## Conclusions

In conclusion, our ex vivo results likely reflect the similar efficacy of glucocorticoids and macrolides to regulate different patterns of inflammation response, innate immunity, and tissue remodeling in CRS and their effects did not vary by the phenotypes of CRS. Our results provide the possibility of using dexamethasone and clarithromycin to reduce the exacerbation of CRS and employing clarithromycin to treat eosinophilic inflammation in CRS as a steroid sparing drug. At same time, our study arouses the need to develop novel therapies targeting Th17 responses in CRS. However, obviously, further in vivo studies are needed to clarify the efficacy of dexamethasone and clarithromycin on the treatment of different phenotypic CRS. Moreover, whether there is a synergetic effect between dexamethasone and clarithromycin on CRS treatment is also an interesting topic for future investigation.

## Methods

### Subjects

In the pilot dose response, cell viability, and dexamethasone receptor blocking study, 10 patients with CRSsNP, 6 patients with non-eosinophilic CRSwNP, and 7 patients with eosinophilic CRSwNP were recruited. In the further comparison study of dexamethasone and clarithromycin, 5 patients with CRSsNP, 6 patients with non-eosinophilic CRSwNP and 6 patients with eosinophilic CRSwNP were enrolled. The detailed clinical data of patients are summarized in Table 1. Diseased ethmoid sinus mucosal tissues and polyp tissues were collected during surgery from CRSsNP and CRSwNP patients, respectively. The diagnosis of CRS was made according to the European Position Paper on Rhinosinusitis and Nasal Polyps [1]. CRSwNP was classified as eosinophilic when percent tissue eosinophils exceeded 10 % of total infiltrating cells as defined by our previous study based on the evaluation of hematoxylin-eosin stained tissue sections [2]. Atopic status was determined using skin prick test with a standard panel of 20 inhalant allergens common in our region [33]. The diagnosis of allergic rhinitis was based on the concordance between a typical history of allergic symptoms and the skin prick test results. The diagnosis of asthma was made according to Global Initiative for Asthma 2006 guideline [34]. Oral glucocorticoid and intranasal steroid spray were discontinued at least 3 months and 1 month before surgery, respectively. The patients who had an acute upper respiratory infection in the 4 weeks before the surgery were excluded in this study. None of subjects had an antrochoanal polyps, cystic fibrosis, primary ciliary dyskinesia, fungal sinusitis, gastroesophageal reflux diseases, aspirin sensitivity, or previous sinus surgery. This study was approved by the Ethics Committee of Tongji Hospital of Tongji Medical College of Huazhong University of Science and Technology and conducted with written informed consents from all participants.

**Table 1:** Clinical data of patients enrolled in pilot and further comparison studies

	Pilot study			Further comparison study		
	CRSsNP	Non-Eos CRSwNP	Eos CRSwNP	CRSsNP	Non-Eos CRSwNP	Eos CRSwNP
Subject, n	10	6	7	5	6	6
Gender, male, n (%)	6 (60 %)	4 (66.7 %)	3 (42.9 %)	3 (60 %)	5 (83.3 %)	4 (66.7 %)
Age (years), median (IQR)	38 (30–47.5)	39 (36.5-52)	35 (26–43.5)	46 (27–49)	35.5 (30.25-41.5)	43 (36.75-53)
Patients with atopy, n (%)	2 (20 %)	0 (0)	1 (14.3 %)	0 (0)	0 (0)	0 (0)
Patients with AR, n (%)	0 (0)	0 (0)	1 (14.3 %)	0 (0)	0 (0)	0 (0)
Patients with asthma, n (%)	0 (0)	0 (0)	0 (0)	0 (0)	0 (0)	0 (0)

CRSsNP, chronic rhinosinusitis without nasal polyps; Non-Eos CRSwNP, non-eosinophilic chronic rhinosinusitis with nasal polyps; Eos CRSwNP, eosinophilic chronic rhinosinusitis with nasal polyps; IQR, inter-quartile range; AR, allergic rhinitis

### Nasal tissue explant culture

Sinonasal mucosal samples were used for ex vivo air-liquid interface culture as described previously [35]. Briefly, the ethmoid mucosa or polyp tissues were sectioned into multiple samples of approximately 6 mm^3^ and sections of tissues were placed on 0.4-μm well inserts (Millipore Corp., Billerica, MA, USA) in 2 mL of Dulbecco’s modified Eagle’s medium/F-12 supplemented with 2 mM glutamine, 100 U/mL penicillin, and 100 μg/mL streptomycin (Invitrogen, Carlsbad, CA, USA) [35, 36]. The tissue samples were oriented with the epithelium being exposed to the air, forming an air-liquid interface to mimic the in vivo situation. The tissues were cultured at 37 °C with 5 % CO_2_ in humidified air. In the pilot dose response and cell viability study, tissues were cultured with dexamethasone or clarithromycin (Sigma, St. Louis, MO, USA) for 24 h, at serial concentrations of 10^−7^ mol/L, 10^−6^ mol/L, and 10^−5^ mol/L. In some experiments with 10^−5^ mol/L of dexamethasone, a glucocorticoid receptor antagonist, mifepristone (10^−5^ mol/L; USBiological, Swampscott, MA, USA), was added to confirm the specific effect of dexamethasone. Based on the results of pilot experiments, in the further comparison study of dexamethasone and clarithromycin, tissues were treated with 10^−5^ mol/L of dexamethasone or clarithromycin for 24 h. This concentration was reported comparable to that seen in serum during oral administration of clarithromycin, and was close to local concentration when glucocorticoids are delivered intranasally, respectively [16, 17]. In tissue explant culture, an equivalent volume of diluent, methanol solution (Sigma) was used as control. After culture, the culture supernatants and tissues were collected and stored at −80 °C for subsequent ELISA and quantitative real-time polymerase chain reaction (PCR) analysis, respectively; or the tissues were subject to cell viability assessment immediately.

### Cell viability assessment

After culture, tissues were harvested and dissociated mechanically with the GentleMACS Dissociator (Miltenyi Biotec Technology & Trading (Shanghai) Co. Shanghai, China) immediately [37]. Then the single cell suspension was generated and the cell viability was estimated by trypan blue (Sigma) dye exclusion test. At least 600 cells were counted in four different fields and the number of viable cells was calculated as a percentage of total cell population [38].

### Quantitative real-time polymerase chain reaction

Total RNA was extracted from tissue samples by using TRI reagent (Invitrogen) and treated by using a DNA-free kit (Fermentas, Hanover, MD, USA) to remove contaminating DNA. One microgram total RNA was reverse-transcribed to cDNA with random hexamer primer as previously described [35, 39]. cDNA equivalent to 40 ng total RNA was used to perform quantitative real-time PCR by using the SYBR Premix Ex Taq kit (TaKaRa Biotechnology, Dalian, China) with appropriate primers as mentioned elsewhere [35, 39]. The following targets were detected, including epithelial-derived mediators, chemokines, pattern recognition receptors (PRRs), tissue remodeling factors, and dendritic cell (DC) markers. The list of genes detected and the corresponding primers are shown in Table 2. Relative gene expression was calculated by using the comparative CT method [35, 39]. Glyceraldehydes-3-phosphate dehydrogenase was used as a housekeeping gene for normalization, and a sample from control group was served as a calibrator. A no template sample was employed as a negative control for PCR assay.

**Table 2:** Primers used for quantitative polymerase chain reaction assay

Primer	Sequence	Annealing temperature (°C)	Expected product size (bp)
CC10	[S] 5'-GGACACACCCTCCAGTTATGA-3'	60	126
	[A] 5'-ATGATGCTTTCTCTGGGCTTT-3'		
IL-33	[S] 5'-ACAGCAAAGTGGAAGAACACAG-3'	60	162
	[A] 5'-CCTTTTGGTGGTTTCTCTCCTA-3'		
TSLP	[S] 5’-CCCAGGCTATTCGGAAACTCA-3’	60	118
	[A] 5'-ACGCCACAATCCTTGTAATTGTG-3'		
IL-25	[S] 5'-AAGGAGATGGTTGGTCAGAAGA-3'	58	182
	[A] 5'-CTCCTAATCGCAAAAGAGCATC-3'		
Osteopontin	[S] 5'-CAACCGAAGTTTTCACTCCAG-3'	58	173
	[A] 5'-ATTCAACTCCTCGCTTTCCAT-3'		
CCL20/MIP-3α	[S] 5'-GAATCAGAAGCAGCAAGCAAC-3'	60	209
	[A] 5'-TTTTTACTGAGGAGACGCACAA-3'		
CXCL19/MIG	[S] 5'-GTTCTTTTCCTCTTGGGCATC-3'	60	108
	[A] 5'-GATAGTCCCTTGGTTGGTGCT-3'		
CCL17/TARC	[S] 5'-GCCCCACTGAAGATGCTG-3'	60	204
	[A] 5'-GCCCTGCACAGTTACAAAAAC-3'		
CCL22/MDC	[S] 5'-TGATTACGTCCGTTACCGTCT-3'	60	179
	[A] 5'-AGTAGGCTCTTCATTGGCTCA-3'		
CXCL5/ENA-78	[S] 5'-TTACAGACCACGCAAGGAGTT-3'	60	105
	[A] 5'-GTTCTTCAGGGAGGCTACCAC-3'		
ECP	[S] 5'-TGCCCTCATAACAGAACTCTCA-3'	60	216
	[A] 5'-GATGGTGGTATCCAGGTGAACT-3'		
MDA-5	[S] 5'-GTTTGGCAGAAGGAAGTGTCA-3'	60	213
	[A] 5'-CTGTAGGGAGGCAGATGATGA-3'		
TLR2	[S] 5'-ATGCTGCCATTCTCATTCTTCT-3'	60	101
	[A] 5'-CTCCAGGTAGGTCTTGGTGTTC-3'		
TLR3	[S]5'-CGCTAAAAAGTGAAGAACTGGA-3'	60	100
	[A]5'-TGAAAACACCCTGGAGAAAACT-3'		
TLR4	[S] 5'-CTTCTCAACCAAGAACCTGGAC-3'	60	158
	[A] 5'-TAGAGAGGTGGCTTAGGCTCTG-3'		
TLR9	[S] 5'-CTACAACCGCATCGTCAAACT-3'	60	217
	[A] 5'-ATTCAGCCAGGAGAGAGAACTG-3'		
EGF	[S] 5'-CTCATCACTGGTTGTGGTTCAT-3'	60	133
	[A] 5'-CATAAAACCTTCACGACACGAA-3'		
MMP9	[S] 5'-ACCACCACAACATCACCTATTG-3'	60	166
	[A] 5'-ACACCAAACTGGATGACGATG-3'		
TGF-β1	[S] 5'-CAGCAACAATTCCTGGCGATA-3'	57	136
	[A] 5'-AAGGCGAAAGCCCTCAATTT-3'		
CD86	[S] 5'-CAGCCTCTTCTTCTCTCAGCAG-3'	60	100
	[A] 5'-CTCACTGGGGTCTGTGGTCT-3'		
OX40L	[S] 5'-AAATGAAGAGGAGCAAGGAGTG-3'	60	247
	[A] 5'-CTGGGAAAGCAAAATGGTAAAG-3'		
ICOSL	[S] 5'-AGGTTTTGAGCGTTGAGGTTAC-3'	60	165
	[A] 5'-GGCTGTTGTCCGTCTTATTGAT-3'		
PD-L1	[S] 5'-GAACTACCTCTGGCACATCCTC-3'	60	126
	[A] 5'-CACATCCATCATTCTCCCTTTT-3'		
CD80	[S] 5'-TAATAAGCAAAGGGAGCACTGG-3'	60	140
	[A] 5'-GCACAGGAGTCTGATGAACAAA-3'		
GAPDH	[S] 5'-ACCCAGAAGACTGTGGATGG-3'	61	201
	[A] 5'-TTCTAGACGGCAGGTCAGGT-3'		

CC10, Clara cell 10-kD protein; IL, interleukin; TSLP, thymic stromal lymphopoietin; CCL, CC chemokine ligand; MIP-3α, macrophage inflammatory protein-3α; CXCL, CXC chemokine ligand; MIG, monokine induced by interferon-γ; TARC, thymus and activation-regulated chemokine; MDC, macrophage-derived chemokine; ENA-78, epithelial neutrophil-activating peptide-78; ECP, eosinophilic cationic protein; MDA-5, melanoma differentiation-associated gene 5; TLR, Toll-like receptor; EGF, epidermal growth factor; MMP9, matrix metalloproteinase 9; TGF-β1, transforming growth factor-β1; OX40L, OX40 ligand; ICOSL, inducible costimulator ligand; PD-L1, programmed cell death ligand 1; GAPDH, glyceraldehydes-3-phosphate dehydrogenase

### ELISA

The protein levels in culture supernatants were measured by using commercial ELISA kits according to the manufacturer’s recommendations. The detected proteins included pro-inflammatory cytokines, Th1/Th2/Th17 cytokines, chemokines, and tissue remodeling factors. The list of proteins detected and the corresponding detection limits are shown in Table 3.

**Table 3:** The lower detection limit for ELISA

Cytokine	Lower detection level (pg/mL)	Source
GM-CSF	8	NeoBioscience Technology Co (Shenzhen, China)
IL-6	0.5	NeoBioscience Technology Co
TNF-α	8	NeoBioscience Technology Co
IL-1β	4	Shanghai Excell Biology, Inc (Shanghai, China)
IFN-γ	0.2	NeoBioscience Technology Co
IL-12	4	eBioscience (San Diego, CA, USA)
IL-4	3.9	Shanghai Excell Biology, Inc
IL-5	2	Shanghai Excell Biology, Inc
IL-13	0.7	eBioscience
IL-17A	0.23	eBioscience
IL-10	0.5	NeoBioscience Technology Co
CXCL10/IP-10	8	NeoBioscience Technology Co
CCL11/eotaxin	1.4	eBioscience
CCL5/RANTES	7	Shanghai Excell Biology, Inc
CXCL8/IL-8	2	eBioscience
VEGF	8	NeoBioscience Technology Co
FGF-basic	7	Shanghai Excell Biology, Inc
PDGF-BB	4.6	eBioscience

GM-CSF, granulocyte-macrophage colony stimulating factor; IL, interleukin; TNF-α, tumor necrosis factor-α; IFN-γ, interferon-γ; CXCL, CXC chemokine ligand; IP-10, interferon-γ-induced protein 10; CCL, CC chemokine ligand; RANTES, regulated upon activation normal T cell expressed and secreted; VEGF, vascular endothelial growth factor; FGF-basic, basic fibroblast growth factor; PDGF-BB, platelet derived growth factor-BB

### Statistical analysis

The data are expressed as median and inter-quartile range or in box plots that represent medians and inter-quartile ranges. Repeated-measures analysis of variance was used to determine a concentration-dependent drug effect on cytokine production and cell viability. The Kruskal-Wallis *H* test was used to assess significant intergroup variability and the Mann–Whitney *U* 2-tailed test was used for between-group comparisons. Significance was accepted at *P* < 0.05.
